# Physician ratings of physician assistant competencies and their experiences and satisfaction working with physician assistants: Results from the supervising physician survey in Ontario, Canada

**DOI:** 10.1177/08404704231173612

**Published:** 2023-05-26

**Authors:** Kristen Burrows, Leslie Nickell, Paul Krueger

**Affiliations:** 1 62703 McMaster University, Hamilton, Ontario, Canada.; 2 12366 University of Toronto, Toronto, Ontario, Canada.

## Abstract

Physician Assistants (PAs) are a relatively new addition to the Ontario healthcare system. To understand the impact of the PA role, this study investigated supervising physician satisfaction and perception of PA roles, interprofessional team integration, pandemic supports, and barriers and enablers to PA employment. A web-based survey was conducted of 118 physician supervisors of Ontario PA education program alumni. PAs were employed in a variety of community and hospital settings. In addition to patient care, PAs were involved teaching (65.6%), quality improvement (52.7%), and mentorship (40.0%). Overall, 92.9% of physicians indicated they were satisfied with their PAs. Important barriers to hiring PAs included maintaining PA salaries, billing limitations, and PA shortages. PAs have established themselves as valuable and competent members of healthcare teams. By continuing to explore the enablers and barriers to PA employment from the physician perspective, health leaders can continue to optimize and support role integration.

## Introduction

The Physician Assistant (PA) role was introduced to Ontario, Canada, in 2007 by the Ministry of Health and Long-Term Care.^1,2,3^ After the success of a provincial pilot project, two PA education programs were launched at McMaster University in 2008, and the University of Toronto in 2010. The goal of PA role integration was to decrease wait times, improve patient access to care, and to provide a cost-effective model for healthcare delivery.^1,2,3^

PAs are trained as generalists within the medical model to allow for a flexible skill set that can be leveraged to fill gaps in the healthcare system. The condensed nature of PA education focuses on history taking, patient assessment, investigations, diagnosis, management, and preventive medicine as defined by the professions National Competency Profile and 12 Entrustable Professional Activities (EPAs).^4^ Upon successful program completion, graduates are eligible to challenge a National Certification Exam through the Physician Assistant Certification Council of Canada. PAs work across a breadth of disciplines and medical sub-specialities: from family medicine, internal medicine, general surgery, and emergency medicine to specialities such as maternal-fetal medicine, hematology, and ophthalmology. PAs work collaboratively with a designated supervising physician to provide direct patient care and are increasingly active in research, quality improvement initiatives, and management roles. They are often referred to as “physician extenders” with a scope of practice defined by the supervising physician.^5^ The PA-physician employment relationship is dependent on the understanding of the supervising physician and PA of the PA’s current competencies and confidence that the PA will seek support when required.^6^

Despite increasing familiarity with the PA role in hospital and community healthcare settings, little research has focused on physician satisfaction and demonstrated PA competencies across the breadth of specialities. As momentum grows for the PA profession, it is essential for health leaders, policy-makers, and health systems administrators to understand and recognize the impact of PA integration into the healthcare system and the relevant impact on patients, physicians, and healthcare teams.^7^ The objectives of this study were to understand the experiences of supervising physicians working with PAs, demonstrate the scope of PA work activity, and to explore physician satisfaction and their rating of PA competencies. By continuing to explore the enablers and facilitators to PA employment from a physician perspective, health leaders and policy-makers can continue to optimize and support role integration, thus ensuring better alignment of limited health human resources.

## Methods

### Setting

Physician Assistant education in Ontario is delivered at the University of Toronto and McMaster University. Both programs are 24 months in duration, with a condensed curricula consisting of a clinical sciences year followed by one year of clinical rotations. In year one, McMaster offers an in-person, problem-based learning approach and the Consortium of PA Education (University of Toronto, Northern Ontario School of Medicine, and the Michener Institute of Education at University Health Network) offers a primarily on-line distributed education model. In year two, both program support core and elective clinical rotations located throughout the province. The province currently supports a total of 54 training seats between both education programs (24 seats at McMaster University, and 30 seats at the University of Toronto). At the time of submission, the Ontario PA education programs have graduated 590 PAs who are working across a broad range of disciplines and in a variety of clinical settings.

### Questionnaire development

The questions included in the survey were developed after a review of the literature (including surveys of supervising physicians of PAs) and a review of the findings from the 2019 PA Alumni Survey.^8^ Questions were generated to better understand supervising physician experiences, perceptions, and satisfaction with their PAs. The research team met frequently to refine the questions, considering issues of reliability, validity, readability, grammar, and appropriateness. The questionnaire was then pretested on a group that included content experts, a research methodologist, and potential respondents. Pretesting helped to identify additional questions to include (or exclude), examine wording of questions, review the format, provide suggestions for implementation, and provide estimates of the time required to complete the survey. The questionnaire was revised based on the pretesting and then formatted into an on-line web-based survey using Qualtrics^XM^. The final draft of the survey was pilot tested and minor revisions were made based on the pilot testing. The questionnaire can be obtained by contacting the corresponding author.

The final questionnaire contained five sections tailored to collecting information on the following: physician experience in supervising a PA; physician perception of work activity supported by a PA; physician satisfaction with the PA role; physician perceptions regarding the role of PAs during the COVID-19 pandemic; and, perceptions regarding past and future barriers and enablers for hiring PAs and general comments.

### Identification of supervising physicians

Ontario PA program alumni (2010-2019) were contacted with a request to provide the contact information (e-mail address) for their current primary supervising physician(s). PA alumni were informed that their supervisor’s participation was voluntary, that all information would remain confidential, and that all responses would later be anonymized. For those who did not feel comfortable providing e-mail contact information, PA alumni were given the option to forward the e-mail request and a direct link to the survey to their supervising physicians. Included with the e-mail to PAs was an on-line link to a one-page survey to collect the contact information of their supervising physicians. This e-mail was followed up by a thank you/reminder e-mail to all PAs, and for those who did not respond to the second request, a final reminder was sent one week later.

### Survey implementation

A modified Dillman approach was used.^9^ All e-mails were personalized and sent jointly from the Ontario PA education program leads. On launch day, each supervising physician was sent an e-mail containing a link to the survey. A thank you /reminder was then sent to all supervising physicians (thanking those who had completed the survey and reminding those who had not, to do so as soon as possible). For those who had still not completed the survey, up to three additional reminder e-mails were sent out weekly.

### Analysis

The data were exported from Qualtrics^XM^ as an SPSS file for analysis. The questionnaire contained several questions with five-point Likert scales. Prior to analysis, the research team reached consensus on the most appropriate way to recode the categorical response data. For example, the five-point Likert scale (excellent, very good, good, fair, and poor) used to assess supervising physicians’ ratings of PAs was recategorized to very good + excellent vs. good + fair + poor. Descriptive statistics were calculated for all variables, including frequency counts, and percentages for categorical variables, or means and standard deviations for continuous variables.

## Results

Of the 468 PAs who were contacted to provide contact information for their supervising physicians, 118 responded. Of the subsequent 118 supervising physicians who were approached to complete the on-line survey, 93 (78.8%) returned a completed questionnaire.

### Supervisory experience and clinical setting

Supervising physicians reported an average of 5.3 years (SD = 3.42) of experience working with their PA(s) and currently supervised on average two PAs (SD = 1.71). Physicians reported supervising PAs in a wide variety of areas including family medicine/primary care (31.2%); general surgery (18.3%); other speciality surgery (18.3%); emergency medicine (17.2%); outpatient medicine (14.0%); mental health (9.7%); other medicine sub-specialities (7.5%); inpatient medicine (5.4%); long-term care or care of the elderly (3.2%); general pediatrics (2.2%); other pediatric specialities (1.1%); and substance use medicine (1.1%). Supervising physicians reported that their PAs clinical work was with an adult population (53.8%), mixed adult/pediatric populations (47.3%), or only pediatrics (8.6%). 92.4% of supervising physicians reported that PAs were fairly or very integrated as part of an interprofessional team. Supervising physicians reported that 87.1% of their PAs were involved in other activities in addition to patient care including teaching (65.6%), quality improvement (52.7%), mentorship (40.0%), research (16.1%), and leadership (15.1%) activities.

### Scope of PA work activity

When asked which activities, in addition to patient care, supervising physicians would like to see their PAs involved in, there was good agreement with activities that PAs were currently involved in including: teaching (67.8%), quality improvement (66.7%), mentorship (46.2%), research (33.3%), and leadership (15.1%) activities. Research and quality improvement were identified as two areas that supervising physicians would like more PA involvement. Overall, 15.1% of supervising physicians indicated they would only like PAs involved in patient care.

### Physician satisfaction and entrustable professional activities

Supervising physicians were asked to rate how well the PAs they currently supervise perform in relation to the professions’ 12 EPAs. The percentage of supervising physicians, who rated the PAs as very good or excellent, ranged from 82.6% to 94.0% (Table 1).

**Table 1.** Supervising physician ratings of PA EPAs.

EPAs	Very good or excellent
**EPA 1:** Practices patient-focused, safe, ethical, professional, and culturally competent medical care across the healthcare continuum. (n = 86)	90.1%
**EPA 2:** Obtains histories and performs physical examinations, demonstrating the clinical judgment appropriate to the clinical situation. (n = 85)	89.4%
**EPA 3:** Formulates clinical questions and gathers required clinical evidence to advance patient care and communicates those results to the patient and medical team. (n = 85)	89.4%
**EPA 4:** Formulates and prioritizes comprehensive differential diagnoses. (n = 86)	82.6%
**EPA 5:** Develops and implements patient-centred, evidence-based treatment plans within the formalized physician, clinical team and caregiver relationship. (n = 86)	86.0%
**EPA 6:** Accurately documents the clinical encounter incorporating the patient’s goals, caregiver goals, decision-making, and reports into the clinical record. (n = 86)	88.4%
**EPA 7**^1^**:** Collaborates as a member of an interprofessional team in all aspects of patient care including transition of care responsibility. (n = 85)	92.9%
**EPA 8**^1^**:** Recognizes a patient requiring immediate care, providing the appropriate management and seeking help as needed. (n = 83)	94.0%
**EPA 9**^1^**:** Plans and performs procedures and therapies for the assessment and the medical management appropriate for general practice. (n = 81)	86.4%
**EPA 10**^1^**:** Engages and educates patients on procedures, disease management, health promotion, wellness, and preventive medicine. (n = 84)	91.7%
**EPA 11**^1^**:** Recognizes and advocates for the patient concerning cultural, community, and social needs in support of positive mental and physical wellness. (n = 83)	91.6%
**EPA 12**^1^**:** Integrates continuing professional and patient quality improvement, life-long learning, and scholarship. (n = 85)	88.2%

^1^Percentages exclude those who selected “Unable to Judge” (1 for EPA7; 3 for EPA8; 5 for EPA9; 1 for EPA10; 2 for EPA11; and 1 for EPA12). Presented percentages represent those who were able to judge.

When asked to rate the overall competency level of the PAs that they currently supervise, 90.4% of supervising physicians rated their competency level as being very good or excellent. Supervising physicians were asked about their level of agreement regarding areas where their PAs have been helpful. Areas of contribution were ranked from high to low based on physician level of agreement (Table 2).

**Table 2.** Supervising physician level of agreement regarding PA contributions.

PA(s) helped…	Agree or strongly agree
With patient charting/documentation (n = 82)	98.8%
With patient education/health promotion (n = 84)	96.3%
With the number of patients seen in clinic/ward per day (n = 83)	95.2%
Improve clinic efficiency (n = 80)	95.0%
To improve patient flow including admissions, length of stay (n = 77)	92.2%
With patient follow-up (n = 81)	91.4%
To improve the quality of care provided to patients (n = 84)	90.5%
Improve patient satisfaction (n = 83)	89.2%
Improve supervisor’s overall well-being (n = 84)	88.1%
To reduce patient wait times (n = 83)	88.0%
With discharge planning (n = 57)	86.0%
With the completion of paperwork such as WSIB and other forms (n = 82)	85.4%
Free up time for supervisor to do non-patient care activities (n = 80)	72.5%
Decrease supervisor’s office/clinic/hospital hours (n = 71)	64.8%

A larger number (91.9%) of supervising physicians agreed or strongly agreed that their PAs have been able to work more independently over time. When asked how satisfied overall they were with the PAs that they currently supervised, 92.9% of supervising physicians indicated they were satisfied or very satisfied. Furthermore, 93.1% indicated that they would likely or very likely recommend that other physicians consider hiring PAs.

### COVID-19 pandemic contributions

Given that this survey was conducted during the COVID-19 pandemic, supervising physicians were also asked to indicate their overall level of agreement about several characteristics demonstrated by their PAs during this time period. Their level of agreement is shown from high to low in Table 3.

**Table 3.** Supervisors’ level of agreement about their PAs during the pandemic.

During the pandemic, my PA(s) demonstrated…	Agree or strongly agree
Adaptability (n = 85)	97.6%
Flexibility (n = 83)	96.4%
Willingness to provide virtual patient care (n = 65)	92.3%
Willingness to be redeployed for pandemic-related activities (n = 59)	86.4%
Willingness to work with COVID-19 patients (n = 73)	76.7%

### Current enablers and barriers

Supervising physicians were asked to rate the importance of several potential enablers and barriers they experienced when hiring their current PA(s). Their ratings are ordered from high to low in Tables 4 and 5 respectively.

**Table 4.** Importance of potential enablers when hiring current PA(s).

Potential enabler	Fairly or very important
Improved patient access to care/improved patient flow (n = 84)	92.9%
Workload distribution or improved work/life balance (n = 84)	91.7%
Ontario PA Career Start Grant (PACS) (n = 79)	73.4%
Previous positive experience working with a PA (n = 63)	73.0%
Hospital financial support (n = 63)	66.7%
Chair/Department support (n = 67)	65.7%
Previous exposure to PA students (n = 71)	57.7%
Practice plan financial support (n = 58)	46.6%
Existing medical directives (n = 77)	40.3%
Clinical space for additional providers (n = 78)	39.7%
Support from other non-physician staff/employees (n = 68)	36.8%

**Table 5.** Importance of potential barriers when hiring current PA(s).

Potential barrier	Fairly or very important
Maintaining PA salary after hiring (n = 87)	85.1%
OHIP billing limitations (n = 83)	55.4%
Concern about PA skill set/competency (n = 87)	43.7%
No PAs available to recruit/hire (n = 86)	39.5%
Institutional resistance (n = 87)	34.5%
Resistance from others (colleagues, management, department) (n = 90)	31.1%
Lack of regulation (n = 86)	29.1%
Issues around role clarity (n = 91)	26.4%
Lack of medical directives (n = 86)	22.1%
Lack of clinical space (n = 89)	19.1%
Site specific limitations (i.e. issues around prescribing, referring) (n = 81)	16.0%
Patient resistance/reluctance to be seen by a PA (n = 89)	12.4%

Almost two-thirds of the supervising physicians (63.6%) indicated that they were fairly or very likely to hire a PA in the next five years. They were asked to rate the importance of several potential barriers to hiring a PA in the future, and the rank order findings of their responses are presented in Table 6.

**Table 6.** Importance of potential barriers when hiring a PA in the future.

Potential barrier	Fairly or very important
Maintaining PA salary after hiring (n = 81)	90.1%
Resources to pay PA salary (n = 84)	88.1%
OHIP billing limitations (n = 73)	56.2%
No PAs available to recruit/hire (n = 80)	50.0%
Concern about PA skill set/competency (n = 81)	42.0%
Lack of regulation (n = 79)	32.9%
Institutional resistance (n = 72)	31.9%
Resistance from others (colleagues, management, department) (n = 76)	27.6%
Lack of medical directives (n = 76)	14.5%
Patient resistance/reluctance (n = 79)	10.1%

## Discussion

### Summary of results

This study has identified key areas where PAs contribute to healthcare in Ontario from the physician perspective, including their perceived competencies and physician satisfaction. The results recognize that patients and physicians benefit from the inclusion of PAs in a broad range of healthcare settings and disciplines. Physicians perceive an increase in patient access to care and improvement in physician work-life balance as important factors when hiring a PA. Physicians endorse the broad skill set and demonstrated medical competencies of PAs and there is strong agreement on what can be entrusted. Given the generalist training of PAs, they are well positioned to fill gaps in the healthcare system and enhance care delivery.^3,6,7,11^ PA role flexibility and adaptability was particularly evident during the COVID-19 pandemic. This study also identified key challenges and barriers to PA employment as perceived by the supervising physicians.

### Explanation of findings

Findings from this study support previous Canadian and international studies that indicate PAs help decrease wait times, improve management and continuity of patient care, improve physician quality of life, and make positive contributions to productivity.^8,10-13^ Previous Ontario studies indicated that barriers to integration included a lack of familiarity with the PA role and scope of practice^3,9^; however, fewer than 30% of current respondents indicated that role clarity was an issue. It is reasonable to expect that as the role continues to be integrated across a region, stakeholders become increasingly familiar with scope of practice and role expectations. Physicians continue to identify lack of sustainable funding models as a barrier to PA hiring, including maintaining a PAs salary after hiring and from provincial health insurance billing limitations regarding shared patient care.^3,7,10^

Our research findings highlight an increased awareness regarding the lack of available PAs to hire, PA role benefits beyond patient care, and direct physician feedback regarding what can be entrusted to a PA. Increased interest and uptake of PAs across the province has created an imbalance between the supply (low number of graduates) and demand (high number of positions), thus making it more challenging for physician employers to add additional PAs to their team. This study also captured new information regarding PA role contributions beyond the provision of direct patient care and Physician interest in supporting opportunities for teaching/mentorship, quality improvement, research, and other leadership activities. Finally, results demonstrated a high level of agreement between physician perception of competencies and the professions EPAs. As noted in other literature, physician feedback regarding PA competencies determines the acceptance, employability, and scope of practice of the employed PA.^5^

### Future directions

Although anecdotal reports indicate high levels of patient satisfaction, understanding the patient experience will be an important component in support of expanding the PA profession. Further study of sustainable funding models will help strengthen PA recruitment and retention across the province and will help inform government health human resource strategic planning regarding physician extender roles. Further exploration is also required to establish sustainable training pathways and research regarding the role of PAs in supporting health services delivery to rural or remote communities.

### Limitations

While supervising physicians indicated that quality of care and patient satisfaction is improved with PA role integration, direct patient feedback was not obtained. This study focused on supervising physician perception but did not include other employer feedback (i.e. medical affairs, clinical managers, or other employers familiar with the PA role). Study findings are limited to Ontario civilian PA education program graduates and do not include Manitoba program graduates, Canadian Armed Forces PAs, or non-Canadian trained PAs. Demographic information related to type of setting (rural, academic, and urban) was not collected and warrants future analysis. This study focused on physician overall perception at the time of survey release and did not differentiate findings based on PA experience, patient satisfaction, or potential financial benefits/costs of supervising a PA. However, this survey was intentionally designed to target the physician and PA interface as a starting point for additional research given that the PA role is dependent on the supervisory relationship with a physician, regardless of the clinical setting.

## Conclusion

PAs are a relatively new profession to the Canadian healthcare landscape, but increased health human resource pressure is driving increased uptake and interest in the role. As evidenced by the results of this survey, PAs have established themselves as competent, collaborative, and valuable members of healthcare teams. As the role expands in other jurisdictions, findings from this study help further define physician perception of PA competencies, role contributions, and overall satisfaction of PA role integration. The establishment of sustainable funding models and education program expansion that reflects physician/health systems demand will help mobilize this flexible and high calibre health professional role.

## References

[1] HealthForceOntario . (n.d.) Physician Assistants: Government of Ontario. Available from: http://www.healthforceontario.ca/en/Home/Health_Providers/Physician_Assistants. Accessed 8 Feb 2019.

[2] Frechette D Shrichand A . Insights into the physician assistant profession in Canada. JAAPA. 2016;29(7):35–9.Va. 10.1097/01.JAA.0000484302.35696.cd 27351645

[3] Vanstone M Boesveld S Burrows K . Introducing physician assistants to Ontario. Health Reform Observer. 2014;2(1).

[4] Jones IW Burrows KE Nickell L Millham A . Canadian physician assistant competency framework 2021. 2021; 3(7).

[5] Chatterjee S Westneat S Wyant A Hunton R . Physicians in Kentucky perceive physician assistants to be competent health care providers. J Physician Assist Educ. 2018;29(4):197-204. 30461585 10.1097/JPA.0000000000000226

[6] Williams LE Ritsema TS . Satisfaction of doctors with the role of physician associates. Clin Med (Lond). 2014;14(2):113-116. 24715119 10.7861/clinmedicine.14-2-113 PMC4953279

[7] Burrows KE Abelson J Miller PA Levine M Vanstone M . Understanding health professional role integration in complex adaptive systems: a multiple-case study of physician assistants in Ontario, Canada. BMC Health Serv Res. 2020;20(1):365. 32349738 10.1186/s12913-020-05087-8 PMC7189743

[8] Burrows KE Nickell L Kreuger P . 2019 physician assistant education program alumni survey. Unpublished.

[9] Dillman DA Smyth JD Christian LM . Internet, Phone, Mail, and Mixed-Mode Surveys: The Tailored Design Method, 4th Edition. Hoboken, New Jersey: John Wiley & Sons, 2014.

[10] Taylor MT Wayne Taylor D Burrows K Cunnington J Lombardi A Liou M . Qualitative study of employment of physician assistants by physicians: benefits and barriers in the Ontario health care system. Can Fam Physician. 2013;59(11):e507-13. 24235209 PMC3828112

[11] Jones IW . How Manitoba Physicians see the value of physician assistants: Manitoba MD views of the value of PAs. JCANPA [Internet]. 2020. 12 [cited 2022 Aug. 2];1(5):11-7.

[12] Hooker RS Everett CM . The contributions of physician assistants in primary care systems. Health Soc Care Community. 2012;20(1):20-31. 21851446 10.1111/j.1365-2524.2011.01021.x PMC3903046

[13] Laurant M Harmsen M Wollersheim H Grol R Faber M Sibbald B . The impact of nonphysician clinicians. Med Care Res Rev. 2009;66(6_suppl):36S-89S. 19880672 10.1177/1077558709346277

